# Correlation between Gene Expression and Osteoarthritis Progression in Human

**DOI:** 10.3390/ijms17071126

**Published:** 2016-07-14

**Authors:** Leilei Zhong, Xiaobin Huang, Marcel Karperien, Janine N. Post

**Affiliations:** Developmental BioEngineering, MIRA Institute for Biomedical Technology and Technical Medicine, Faculty of Science and Technology, University of Twente, P.O. Box 217, 7500 AE Enschede, The Netherlands; l.zhong@utwente.nl (L.Z.); x.huang-1@utwente.nl (X.H.); h.b.j.karperien@utwente.nl (M.K.)

**Keywords:** osteoarthritis, OARSI grading, gene expression, cartilage degeneration, hypertrophy

## Abstract

Osteoarthritis (OA) is a multifactorial disease characterized by gradual degradation of joint cartilage. This study aimed to quantify major pathogenetic factors during OA progression in human cartilage. Cartilage specimens were isolated from OA patients and scored 0–5 according to the Osteoarthritis Research Society International (OARSI) guidelines. Protein and gene expressions were measured by immunohistochemistry and qPCR, respectively. Terminal deoxynucleotidyl transferase dUTP nick end labeling (TUNEL) assays were used to detect apoptotic cells. Cartilage degeneration in OA is a gradual progress accompanied with gradual loss of collagen type II and a gradual decrease in mRNA expression of *SOX9*, *ACAN* and *COL2A1*. Expression of WNT antagonists *DKK1* and *FRZB* was lost, while hypertrophic markers (*RUNX2*, *COL10A1* and *IHH*) increased during OA progression. Moreover, *DKK1* and *FRZB* negatively correlated with OA grading, while *RUNX2* and *IHH* showed a significantly positive correlation with OA grading. The number of apoptotic cells was increased with the severity of OA. Taken together, our results suggested that genetic profiling of the gene expression could be used as markers for staging OA at the molecular level. This helps to understand the molecular pathology of OA and may lead to the development of therapies based on OA stage.

## 1. Introduction

Osteoarthritis (OA) is a multifactorial disease of the joints, affecting many parts of the joint, including bone, synovium, ligaments and articular cartilage (AC). It is characterized by the progressive destruction of the articular cartilage matrix [1]. Cartilage damage in OA is likely to result from the aggregate effect of multiple genetic, environmental, mechanical and cell biological factors driving changes in gene expression [2].

The pathology of OA is complex; the underlying mechanism behind OA development and progression is still unknown. Many studies of OA progression are based on animal models [3,4,5] that may not be translatable into human disease and therapy. Although gene expression has been studied in normal and advanced OA [6,7], little is known about what happens in transition stages during OA progression. Studies about gene expression in different steps of OA based on OA scores in human cartilage have not been reported and may provide clues for comprehensive understanding of the progression of OA. This would mean that if we measure the expression of these genes in any given patient, we may tailor therapy based on the stage of OA of the individual patient.

It has been shown that the cartilage-specific transcription factor sex-determining region Y box 9 (SOX9) expression is decreased at the mRNA and protein levels in OA cartilage [8]. Collagen type II, α1 (COL2A1) is reduced, while collagen type I, α1 (COL1A1) is increased during the progression of human OA [8,9]. The loss of cartilage markers is not the only known characteristic of OA, as derailed hypertrophic differentiation in AC has been implicated in the pathogenesis of OA, at least in a subset of patients [10]. We have previously shown that dickkopf 1 homolog (DKK1), frizzled-related protein (FRZB) and bone morphogenetic protein (BMP) antagonist gremlin1 (GREM1) as antagonists of the wingless-type MMTV integration site (WNT) or bone morphogenetic protein (BMP)-signaling pathways, respectively, are key factors in controlling the articular chondrocyte phenotype. In addition, creating permanent cartilage under hypoxia conditions correlates with the expression of these three natural antagonists [11].

It has been indicated that runt-related transcription factor 2 (RUNX2), is a positive regulator for chondrocyte maturation [12] and that it is highly expressed in OA cartilage as compared to normal cartilage [13]. Collagen type X, α1 (COL10A1) is a typical hypertrophic marker and a direct transcriptional target of RUNX2 [14]. Upregulation of COL10A1 expression is observed in human OA cartilage [15]. Indian hedgehog (IHH) is mainly produced by prehypertrophic chondrocytes and regulates chondrocyte hypertrophic differentiation [16]. *IHH* and matrix metallopeptidase 13 (*MMP13*), are upregulated in human OA and are correlated with OA progression [17]. *BMP2* and the canonical WNT target gene *AXIN2* are upregulated in injured human articular cartilage explants [18]. Although expression of various genes has been reported in OA, as mentioned above, very little is known about the expression of these genes at different stages of OA and the correlation with OA severity.

In order to investigate the cellular changes and the changes in gene expression that are directly involved in cartilage degeneration, we performed IHC to detect the expression of important proteins. Quantitative polymerase chain reaction (qPCR) assays were used to quantify the expression of OA-related genes: the cartilage markers: *SOX9*, *ACAN* and *COL2A1*; WNT antagonists: *DKK1*, *FRZB* and *GREM1*; hypertrophic markers: *RUNX2*, *COL10A1* and *IHH*; *AXIN2*, *BMP2* and a dedifferentiation marker *COL1A1*. Finally, we investigated the correlation between gene expression, apoptosis and the severity of OA.

This is, to our knowledge, the first comprehensive study determining the correlation between the expression of different major signal transduction factors and apoptosis and different stages of OA in human. Based on our data, the specific gene or protein expression can be coupled to the stage of OA disease, which may ultimately improve OA diagnosis and treatment.

## 2. Results

### 2.1. During OA Progression, the Expression of COL2A1 Decreases, while the Expression of GREM1 and COL10A1 Increases

**Figure 1:**
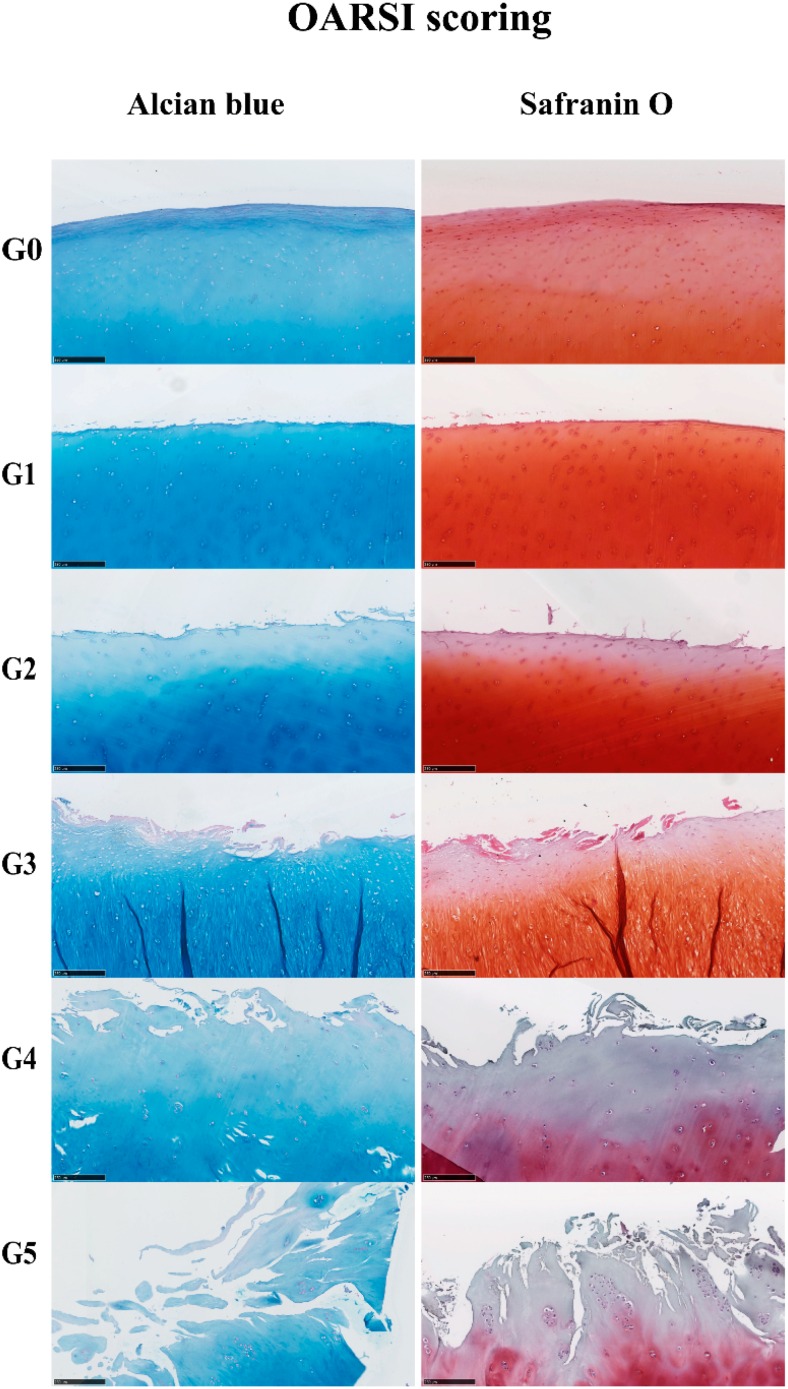
Histological changes of OA cartilage. Five micrometer paraffin sections of cartilage stained by Alcian blue and Safranin O (scale bar 250 µm). The severity of the OA lesion was graded on a scale of 0–5.

**Figure 2:**
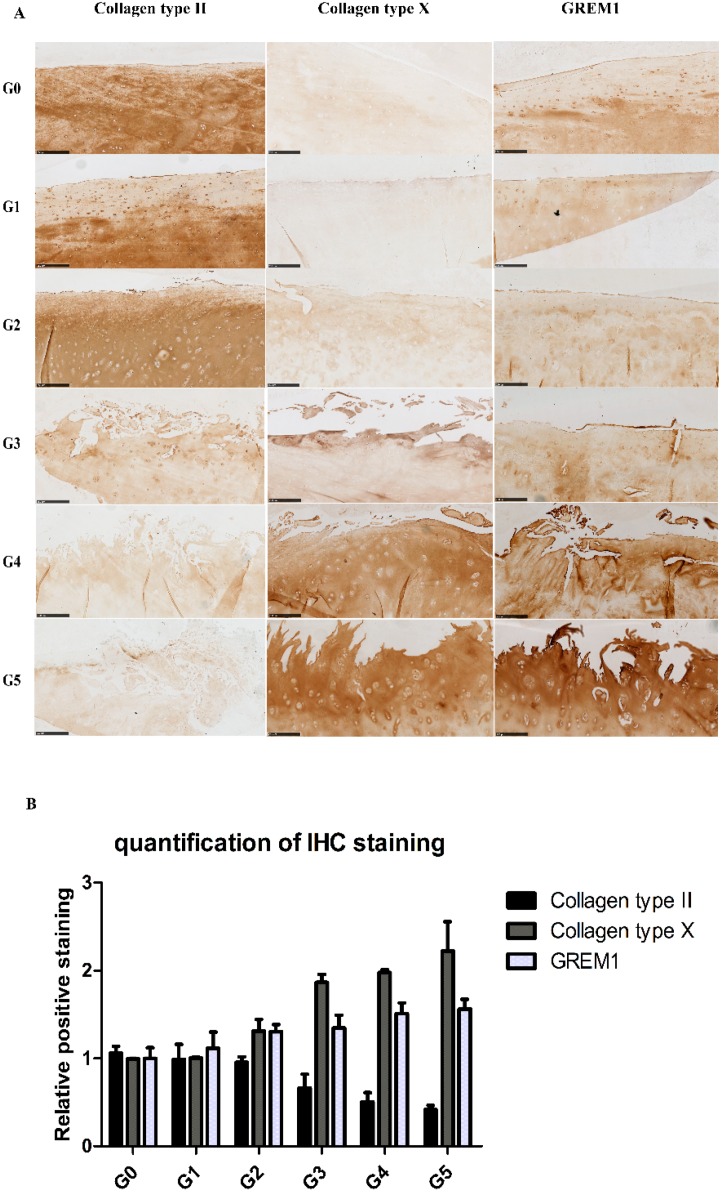
The protein expression of collagen type II, collagen type X and GREM1 was visualized by IHC (scale bar 250 μm). (**A**) Representative pictures are shown. Images were taken using the Nanozoomer. G0, G1, G2, G3, G4, G5 = Grade 0, Grade 1, Grade 2, Grade 3, Grade 4, Grade 5; (**B**) Quantification of positive staining was performed by ImageJ software (Wayne Rasband, Bethesda, MD, USA).

Cartilage samples showed microscopic changes, mostly related to the gradual reduction of Alcian blue and Safranin O staining and the loss of cartilage integrity with the severity of OA (Figure 1). Based on the histological features, the severity of OA was represented by scores from 0–5 by assessing structural damages and cellular abnormalities according to the OARSI guidelines [19]. From OARSI Grades 0–5, cartilage showed histological changes, from intact and smooth surfaces to discontinued surfaces, from fissures in the superficial layer to fissures extending into the deep layers and from slight loss to extensive loss of Alcian blue and Safranin O staining (Figure 1). The IHC results (Figure 2A,B) confirmed the loss of collagen type II with increasing severity of OA; the expression of collagen type II was measured in each patient (Figure S1). GREM1 and collagen type X were increased in high-grade OA; the expression of GREM1 and collagen type X was measured in each patient (Figures S2 and S3). Statistical analyses of IHC quantification indicated that the expression of collagen type II (*r* = −0.972, *p* = 0.001) was negatively correlated with OA grading, while collagen type X (*r* = 0.972, *p* = 0.001) and GREM1 (*r* = 0.987, *p* = 0.0002) were positively correlated with OA severity.

### 2.2. Gene Expression Profiles in Cartilage at Different Stages

To characterize the gene expression during OA progression, qPCR was performed in cartilage specimens with Grades 0, 1, 2, 3, 4 and 5. The expression of cartilage-related markers is summarized in Table 1. For all three cartilage markers, *SOX9*, *ACAN* and *COL2A1*, gene expression gradually decreased with increasing severity of OA. This was especially obvious for the expression of *SOX9*, the master transcription factor for chondrocyte development [20,21], which was reduced to below detection levels in Grade 5 tissue. The expression of the chondrocyte dedifferentiation marker *COL1A1* gradually increased from Grades 2–5. The ratio *COL2A1*/*COL1A1* was sharply decreased from Grades 0–5.

Previously, we have shown that WNT antagonists DKK1 and FRZB and the BMP antagonist GREM1 are regulators for cartilage homeostasis [10]. In our study, the expression of *DKK1* was significantly decreased at Grades 3, 4 and 5; *FRZB* was linearly decreased from Grades 1–4. The expression of *GREM1* slightly decreased between Grades 0 and 2 and steeply increased between Grades 4 and 5. This increase was further substantiated by an increase in GREM1 staining in IHC (Figure 2 and Figure S2).

In a subset of patients, OA is associated with hypertrophic differentiation of chondrocytes [22]. The expression of hypertrophy-related genes is summarized in Table 2. The transcription factor linked to chondrocyte hypertrophy, *RUNX2*, was under the detection level at Grades 0 and 1, which was gradually increased from Grade 2 with the severity of OA. *RUNX2* drives the expression of the terminal differentiation markers, including *COL10A1* and *IHH*, which were increased 10- and nine-fold, respectively, at Grade 5. *COL10A1*, which was under the detection level at Grades 0–3, was first detected at Grade 4. The gene expression is in line with the IHC results (Figure 2 and Figure S3).

Since WNT and BMP signaling are indicated to be involved in cartilage pathophysiology [23], we measured the expression of *AXIN2* as a target gene of WNT signaling and *BMP2* as a target gene of BMP signaling. *AXIN2* was not detectable in cartilage Grades 0, 1, 2 and 3; however, it started to be expressed in OA Grade 4 and increased further at Grade 5. The expression of *BMP2* was sharply increased at Grade 2, further increased at Grade 3 and could not be detected at Grades 4 and 5.

### 2.3. Correlation between Gene Expression and the Severity of OA

The Pearson correlation method was applied to reveal a correlation between gene expression based on qPCR data and OA severity. The expression of *SOX9* (*r* = −0.927, *p* = 0.008), *ACAN* (*r* = −0.959, *p* = 0.002) and *COL2A1* (*r* = −0.960, *p* = 0.002) was negatively correlated with OA, while the expression of the chondrocyte dedifferentiation marker *COL1A1* (*r* = 0.963, *p* = 0.002) was positively correlated with OA grading (Table 1).

Previously, we have shown that the expression of these *DKK1*, *FRZB* and *GREM1* is downregulated in OA [10]. We therefore looked at the correlation between *DKK1*, *FRZB* and *GREM1* expression and OA progression (Table 1). *DKK1* (*r* = −0.812, *p* = 0.05) and *FRZB* (*r* = −0.896, *p* = 0.016) mRNA expression levels were negatively correlated with the grading of knee OA. Although high expression of GREM1 was observed in high-grade OA (Grades 4 and 5) by qPCR and IHC, we found a moderate correlation of *GREM1* with OA grading*,* which was not significant, probably due to the relatively small sample size (*r* = 0.714, *p* = 0.111). However, the expression of *RUNX2* (*r* = 0.908, *p* = 0.012) and *IHH* (*r* = 0.961, *p* = 0.0018) showed a significantly positive correlation with OA grading (Table 2).

In addition, we found that *FRZB* was positively correlated with cartilage marker *ACAN* (*r* = 0.944, *p* = 0.005) and *COL2A1* (*r* = 0.868, *p* = 0.03); *IHH* was negatively correlated with all three cartilage markers *SOX9* (*r* = −0.968, *p* = 0.001), *ACAN* (*r* = −0.914, *p* = 0.011) and *COL2A1* (*r* = −0.914, *p* = 0.011); *FRZB* was positively correlated with *DKK1* (*r* = 0.816, *p* = 0.05); and *GREM1* was positively correlated with the hypertrophic markers *COL10A1* (*r* = 0.995, *p* = 0.000035), *IHH* (*r* = 0.829, *p* = 0.041), *RUNX2* (*r* = 0.997, *p* = 0.003) and *AXIN2* (*r* = 0.933, *p* = 0.007). We did not observe a significant correlation between the expression of the antagonists and the tested hypertrophic markers (Table S1).

### 2.4. Chondrocyte Apoptosis Is Associated with the Severity of OA

**Figure 3:**
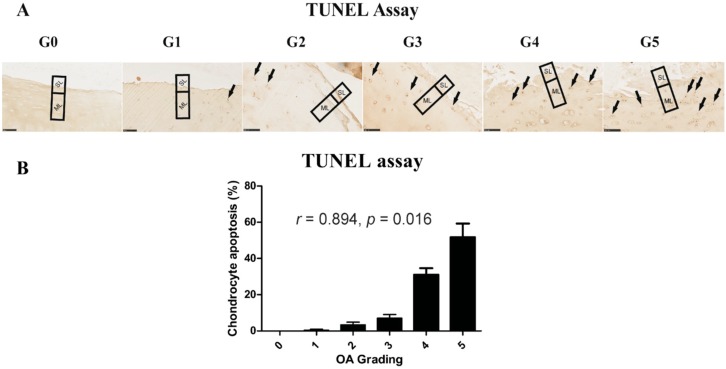
Correlation between histopathological grade and chondrocyte apoptosis. (**A**) The TUNEL assay was performed to identify chondrocyte apoptosis at different stages of OA (scale bar: 100 μm). Arrows indicate apoptotic cells; (**B**) Apoptotic cells were counted in the superficial layer (SL) and middle layer (ML) in cartilage sections for quantification. Apoptosis is quantified as the percentage apoptotic cells with respect to the total cells counted.

It has been indicated that death of chondrocytes and the loss of extracellular matrix are key features in cartilage degeneration during OA [24]. We therefore performed TUNEL staining for detecting DNA fragmentation in different stages of OA (Figure 3). It has been reported that chondrocyte apoptosis is observed in both the superficial and middle layer in cartilage [25]. For quantification, the number of apoptotic chondrocytes was counted in the superficial (SL) and middle layers (ML). The percentage of apoptotic chondrocytes at higher stages of OA (Grades 4 and 5) was significantly higher than that in the lower stages of OA (Grades 1 and 2); apoptotic cells were not observed at Grade 0. There was a significant positive correlation between grading and apoptotic chondrocyte numbers in OA cartilage (*r* = 0.894, *p* = 0.016).

## 3. Discussion

In this study, cartilage samples from patients were graded according to OARSI guidelines and varied from Grades 0–5. Twelve candidate cartilage-related genes were quantified by qPCR and/or immunohistochemistry.

It is important to discuss the fact that when we obtain cartilage samples from OA patients undergoing total joint replacement surgery, the articular cartilage in the joint from most of the patients is almost gone. In addition, cartilage with multiple OA grades is found within single joints. RNA samples from one patient therefore are not enough for any genetic study, such as ours, so we pooled cartilage samples with the same OA grade (according to the OARSI guidelines) from different patients (Table S2). We therefore can only discuss the trend of the gene expression levels coupled to the OA grade of the cartilage. For the qPCR assays, we have no information on the patient-to-patient variability, but results are an average of the material of multiple patients. It is therefore key to note that the protein expression levels of COL2A1, COL10A1 and GREM1, as measured in IHC experiments and which were done for each patient, correspond to the trend described with the qPCR experiments.

Chondrocytes express specific markers, such as collagen type II and the chondrogenic master regulator, the transcription factor, SOX9 [21]. Damage to collagen type II and loss of other cartilage ECM components occur in OA [26,27]. As expected, the gene expression of all three chondrocyte markers, *SOX9*, *ACAN* and *COL2A1*, was decreased with OA severity and negatively correlated with OA severity in line with previous reports [28]. Interestingly, while the expression of *ACAN* and *COL2A1* was sharply decreased between Stages 2 and 5, the loss of *SOX9* was more gradual. This indicates the lack of a one to one relationship between *SOX9* and these cartilage markers despite the fact that these markers are direct target genes of the transcription factor *SOX9*. This may imply that OA is initiated by differential regulation of the signaling network that regulates SOX9 protein activity and not with downregulation of *SOX9* at the transcriptional level. The loss of expression of COL2A1 also was confirmed by IHC, which was in accordance with previously-described data [29,30].

COL1A1 is a fibroblastic marker expressed in dedifferentiated chondrocytes [31,32]. We found that *COL1A1* expression increased significantly in higher OA grades and that the ratio of *COL2A1*/*COL1A1* dropped strongly with OA severity. This expression trend is in agreement with previous reports that collagen I was strongly increased at end-stage OA as compared to normal and early-stage OA [27,33] and that a shift of phenotypes towards fibroblasts was indicated by the drop in the *COL2A1*/*COL1A1* ratio in OA [34,35].

Previously, we reported that three antagonists, DKK1, FRZB and GREM1, are key factors that maintain cartilage homeostasis by diminishing terminal hypertrophic differentiation in long-bone explant cultures and chondrogenically-differentiating human mesenchymal stem cells (hMSCs) [10]. DKK1 is associated with OA development, and high levels of DKK1 have a protective function against cartilage degeneration [36,37,38]. FRZB-knockout mice displayed severe OA cartilage degeneration in a mouse model of joint instability, enzymatic injury or inflammation [39]. In addition, it was shown that the highest FRZB serum levels were associated with a modest reduction in the risk of the incidence of hip OA [36]. We found that the expression of *DKK1* and *FRZB* was lost with the increased severity of OA and that both of these two factors were negatively correlated with OA grading. This is in line with the proposed protective roles of DKK1 and FRZB in articular cartilage and that the loss of these factors might lead to OA progression. We found downregulation of *GREM1* in low-grade OA, while the expression is upregulated in advanced OA. IHC demonstrated that GREM1 was highly expressed in late-stage OA, which has been reported [7]. Our group previously demonstrated that *GREM1* mRNA expression was decreased in OA [22]. In this previous study, the decrease in *GREM1* mRNA expression was measured between macroscopically healthy looking cartilage from an OA joint and OA cartilage without grading of samples. The samples from the previous study might belong to Grades 0–2. However, in the current study, we used histological grading of the samples from OA Grades 0–5, thereby being more careful in specifying healthy and OA tissue. In our previous study, we also found the increase in *GREM1* expression specifically after mechanical loading [22]. In the present study, we did not identify whether the high-grade OA cartilage was isolated from load-bearing regions, which may explain the high expression of GREM1 in end-stage OA. However, we cannot exclude that the differences are due to joint-specific differences in gene expression.

Hypertrophy-like changes of chondrocytes have been reported in both human OA joints and experimental models of OA [40,41,42]. These changes play a crucial role in the OA disease progress since they result in protease-mediated cartilage degradation [43]. When chondrocytes in OA undergo hypertrophic differentiation, this changes their behavior, as indicated by the increased expression of hypertrophic markers, matrix calcification and an enlarged cellular volume [44]. In this study, the expression of three hypertrophic markers, *RUNX2*, *COL10A1* and *IHH,* gradually increased with the severity of OA. In addition, *RUNX2* and *IHH* positively correlated with OA severity. *COL10A1* was not expressed in healthy articular cartilage (Grade 0) and in early stage of OA (Grades 1, 2 and 3), but highly expressed in late stage OA (Grades 4 and 5). These results are in line with another finding that collagen type X is consistently found in advanced stages of OA and is absent in normal cartilage [45], indicating that hypertrophic differentiation might play a role in late-stage OA.

It has been shown that WNT [46,47] and BMP [48] are involved in experimental and human OA [49]. In our study, the target gene of canonical WNT signaling, *AXIN2*, became highly detectable at the mRNA level in OA Grades 4 and 5. Excessive activity of WNT signaling increases chondrocyte hypertrophic differentiation and correlated with the expression of matrix-degrading enzymes [43]. This high activity of WNT signaling at late stages of OA might be related to the reduced expression level of the WNT antagonists *DKK1* and *FRZB* at the mRNA level, leading to high expression of hypertrophic markers, subsequently contributing to OA. *BMP2* expression was below the detection levels in normal cartilage (Grade 0) and was low in OA Grade 1, but strongly increased at Grade 2 and further increased at Grade 3, followed by undetectable levels in Grades 4 and 5. Similar results are observed in previous studies showing that *BMP2* mRNA expression and protein expression are low in normal cartilage, but are increased in the area directly around OA lesions and in OA cartilage [50,51]. The regulation of BMP2 varies in acute OA and chronic OA. In chronic OA, patients a reduced BMP2 expression is found [52]. The absence of *BMP2* expression in Grades 4 and 5 in our study may indicate that the patients have chronic OA.

Chondrocytes are the only cell type present in cartilage and are responsible for the maintenance of the specialized ECM of the tissue [53]. During the development or progression of OA, chondrocytes change their function or undergo apoptosis or chondroptosis. It has been reported that in human OA articular cartilage, the main pathway of chondrocyte death is apoptosis [54], and that apoptosis mainly occurred in the superficial and middle layers in equine OA. In our study, the number of apoptotic cells was significantly increased in the superficial and middle layer in high-grade OA, but not in low-grade OA. Whether chondrocyte apoptosis is a cause or a result of cartilage degeneration in OA is debatable. Our results, however, show that the apoptosis of chondrocytes correlates with cartilage degeneration and, as such, might play an important role in the pathogenesis of OA.

## 4. Materials and Methods

### 4.1. Cartilage Samples Collection

The collection and use of human cartilage was approved by the local hospital ethical committees, and for all samples, informed written consent was obtained. Cartilage specimens were isolated from 12 patients (mean age ± SD: 68 ± 6 years) with OA undergoing total knee replacement surgery (Table S2). In order to get comparable cartilage samples for histology and RNA extraction, several cartilage pieces were removed from the same joint (Figure S4), and for each of the specimens, a cartilage score was determined. Each cartilage specimen was cut into 2 equal parts. One half was used for histology for grading of OA; the other half was used for RNA isolation and qPCR analysis. For RNA isolation, subchondral bone was removed from the cartilage, and samples were cut into small pieces (1–2 mm) and quickly snap frozen in liquid nitrogen (LN_2_). The frozen samples were stored at −80 °C until use.

### 4.2. Histological Analysis

Cartilage samples were collected into 10-mL tubes, washed twice with PBS and fixed using 10% phosphate-buffered formalin (pH = 7.0, Sigma Aldrich, St. Louis, MO, USA) overnight at 4 °C. In the next step, samples were decalcified for 4 weeks in 12.5% (*w*/*v*) EDTA solution containing 0.5% phosphate-buffered formalin (pH = 8.0). After decalcification, samples were dehydrated using graded ethanol and embedded in paraffin.

### 4.3. Safranin O and Alcian Blue Staining

A microtome (Thermo Scientific Shandon, Illkirch Cedex, France) was used to cut 5 μm-thick sections. Sections were then deparaffinized in xylene and hydrated using a graded ethanol series. Slides were either stained for sulfated glycosaminoglycans (GAG) with a 0.1% solution of Safranin O dissolved in distilled water for 5 min and counterstained with hematoxylin dissolved in water to visualize nuclei or stained for sulfated GAG with a 0.5% solution of Alcian blue (pH = 1.0, adjusted with HCL) for 30 min and counterstained with nuclear fast red to visualize nuclei as described previously [55].

### 4.4. Immunohistochemistry

Immunohistochemical staining of COL2A1, COL10A1 and GREM1 was performed on 5-μm tissue sections, which were pre-incubated with 5 μg/mL proteinase K for 10 min in Tris-EDTA (TE) buffer at room temperature (RT) followed by incubation with 1 mg/mL hyaluronidase dissolved in PBS for 40 min at 37 °C. Rabbit polyclonal collagen type II (Abcam, ab34712, Cambridge, UK) and rabbit polyclonal GREM1 (Santa Cruz biotechnology, Dallas, TX, USA) were diluted 1:200; mouse monoclonal antibody against collagen type X (Quartett biochemicals, Berlin, Germany) was diluted 1:100 in PBS containing 1.5% normal goat blocking serum and incubated overnight at 4 °C. Non-immune controls underwent the same procedure without primary antibody incubation. The biotinylated secondary antibody was diluted 1:200 in normal goat blocking serum and incubated for 30 min at RT. Staining was developed using a rabbit or mouse ABC staining system according to the manufacturer’s protocol and imaged using a Nanozoomer (Hamamatsu Photonics, Hamamatsu, Japan).

### 4.5. RNA Isolation and qPCR

Cartilage pieces of samples with the same OA grade from different patients were pooled in a pre-cooled Cryo-Cup Grinder for crushing. The obtained cartilage powder was collected into 50-mL tubes, and samples were weighed. One milliliter of TRIZOL reagent (ThermoFisher scientific, Waltham, MA, USA) per 50–100 mg sample was added. Total RNA was isolated from the lysate according the manufacturer’s protocol. The precipitated RNA was dissolved in RNase-free water and subsequently treated with RNase-free DNase I (Invitrogen life technologies, Carlsbad, CA, USA). The concentration of RNA was measured using the Nanodrop 2000. cDNA was obtained from 1 μg of RNA with a cDNA synthesis kit (Bio-Rad, Hercules, CA, USA). qPCR was performed using the SYBR Green sensimix (Bioline, London, UK). PCR reactions were carried out using the Bio-Rad CFX96 under the following conditions: cDNA was denatured for 5 min at 95 °C, followed by 39 cycles consisting of 15 s at 95° C, 15 s at 60 °C and 30 s at 72 °C. For each reaction, a melting curve was generated to test primer dimer formation and non-specific priming. Gene expression was normalized using GAPDH as the housekeeping gene. Primer sequences are listed in Table S3.

### 4.6. Apoptosis Assay

The apoptosis of cells was detected in paraffin-embedded tissues using The DeadEnd™ colorimetric TUNEL assay (Promega, Madison, WI, USA) following the manufacturer’s procedure. Apoptotic nuclei were stained dark brown.

### 4.7. Statistical Analysis

Data are expressed as the mean. Correlations between the gene expression levels with severity of OA were assessed using Pearson’s correlation analysis. *p* < 0.05 was considered statistically significant.

## 5. Conclusions

We have shown that the expression of cartilage-related genes (*SOX9*, *ACAN*, *COL2A1*, *DKK1*, *FRZB*) was decreased while that of hypertrophy- or OA-related genes (*RUNX2*, *COL10A1*, *COL1A1*, *IHH*, *AXIN2*) was increased or could be detected during OA progression. Moreover, we see a sharp loss of the *SOX9* target genes, while the loss of *SOX9* mRNA expression is more gradual, suggesting that the loss of *COL2A1* and *ACAN* during OA might be also regulated by other additional mechanisms. We therefore conclude that the expression of cartilage-specific genes correlates negatively with the different stages of OA, and hypertrophy genes correlate positively with OA severity. In addition, at high OA grades, apoptosis was induced.

These differences in gene expression provide us with an OA stage-specific gene expression profile that can be used for staging OA at the molecular level and ultimately to therapeutic targeting of chondrocyte hypertrophy by the WNT signaling antagonists DKK1 and FRZB.

## Figures and Tables

**Table 1:** Expression of cartilage-related genes in OA cartilage and correlation with the severity of OA (*n* = 12). RT-PCR was performed to assess gene expression. Pearson correlation was used to examine the correlation between gene expression and the severity of OA. *p* < 0.05 was considered statistically correlated. ns: no correlation; * *p* < 0.05, ** *p* < 0.01: significant correlation.

Gene Name	Protein Function	Gene Expression Trend	Correlation between Gene Expression and OA Severity		Correlation Significant
*SOX9*	Chondrogenic transcription factor for chondrogenesis	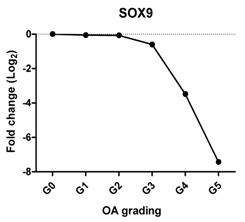	Pearson Correlation Coefficients (*r*)	*p*-Values	**
			−0.927	0.008	
*ACAN*	Extracellular matrix protein, provides strength to cartilage	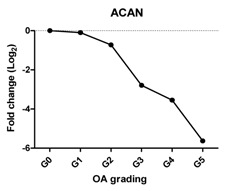	−0.959	0.002	**
*COL2A1*	Extracellular matrix protein, provides cartilaginous framework and tensile strength	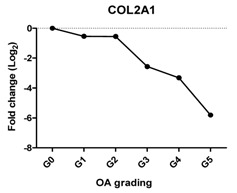	−0.960	0.002	**
*COL1A1*	Provides cartilaginous framework, the marker of dedifferentiated chondrocyte	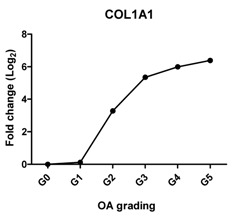	0.963	0.002	**
*Col2A1*/*COL1A1*	Reflects the replacement of collagen type II by collagen type I during OA	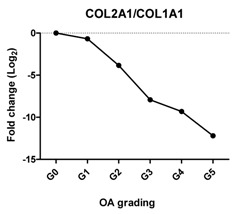	−0.868	0.025	*
*DKK1*	Blocks chondrocyte hypertrophy, promotes chondrogenesis	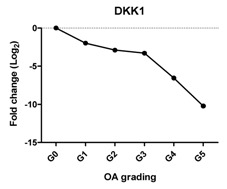	−0.812	0.050	*
*FRZB*	Inhibits chondrocyte hypertrophy, promotes chondrogenesis	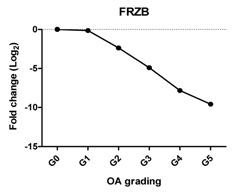	−0.896	0.016	*
*GREM1*	Inhibits terminal chondrocyte differentiation and endochondral bone formation	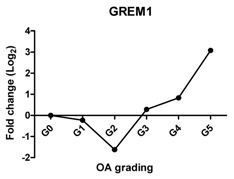	0.714	0.111	ns

**Table 2:** Expression of hypertrophy-related genes in OA cartilage and correlation with the severity of OA (*n* = 12). RT-PCR was performed to assess gene expression. Pearson correlation was used to examine the correlation between gene expression and the severity of OA. *Y* axis = 0, indicated by #, means this gene is under the detection level. *p* < 0.05 was considered statistically correlated. ns: no correlation; * *p* < 0.05, ** *p* < 0.01: significant correlation.

Gene Name	Protein Function	Gene Expression Trend	Correlation between Gene Expression and OA Severity		Correlation Significant
*RUNX2*	Promotes chondrocyte hypertrophy and bone formation	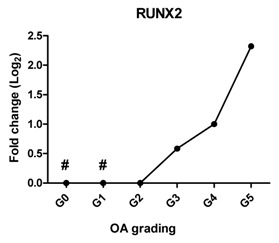	0.908	0.012	*
*COL10A1*	The marker of hypertrophic chondrocytes, regulates matrix mineralization	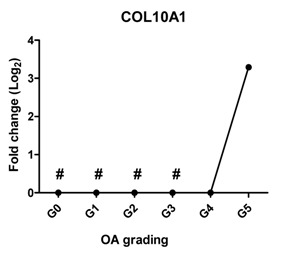	0.705	0.118	ns
*IHH*	Promotes chondrocyte hypertrophy and bone formation	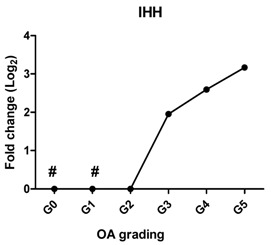	0.961	0.002	**
*AXIN2*	Induces chondrogenesis at low level while inhibits chondrogenic differentiation at high level	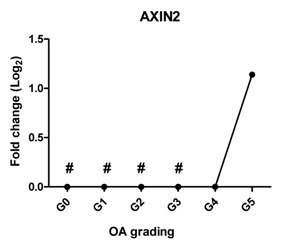	0.827	0.052	ns
*BMP2*	Stimulates chondrogenesis and increases cartilage matrix turnover	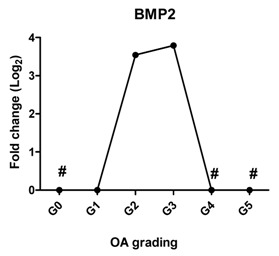	−0.007	0.990	ns

## Supplementary Materials

Supplementary materials can be found at http://www.mdpi.com/1422-0067/17/7/1126/s1.

## References

[1] Felson D.T., Zhang Y., Hannan M.T., Naimark A., Weissman B.N., Aliabadi P., Levy D. (1995). The incidence and natural history of knee osteoarthritis in the elderly. The Framingham Osteoarthritis Study. Arthritis Rheum..

[2] Goldring M.B., Goldring S.R. (2007). Osteoarthritis. J. Cell. Physiol..

[3] Altman R.D., Dean D.D. (1990). Osteoarthritis research: Animal models. Semin. Arthritis Rheum..

[4] Bendele A., McComb J., Gould T., McAbee T., Sennello G., Chlipala E., Guy M. (1999). Animal models of arthritis: Relevance to human disease. Toxicol. Pathol..

[5] Bendele A.M. (2001). Animal models of osteoarthritis. J. Musculoskelet. Neuronal. Interact..

[6] Ramos Y.F., den Hollander W., Bovee J.V., Bomer N., van der Breggen R., Lakenberg N., Keurentjes J.C., Goeman J.J., Slagboom P.E., Nelissen R.G. (2014). Genes involved in the osteoarthritis process identified through genome wide expression analysis in articular cartilage; the RAAK study. PLoS ONE.

[7] Tardif G., Hum D., Pelletier J.P., Boileau C., Ranger P., Martel-Pelletier J. (2004). Differential gene expression and regulation of the bone morphogenetic protein antagonists follistatin and gremlin in normal and osteoarthritic human chondrocytes and synovial fibroblasts. Semin. Arthritis Rheum..

[8] Kim K.I., Park Y.S., Im G.I. (2013). Changes in the epigenetic status of the SOX-9 promoter in human osteoarthritic cartilage. J. Bone Miner. Res..

[9] Miosge N., Hartmann M., Maelicke C., Herken R. (2004). Expression of collagen type I and type II in consecutive stages of human osteoarthritis. Histochem. Cell Biol..

[10] Leijten J.C., Emons J., Sticht C., van Gool S., Decker E., Uitterlinden A., Rappold G., Hofman A., Rivadeneira F., Scherjon S. (2012). Gremlin 1, frizzled-related protein, and Dkk-1 are key regulators of human articular cartilage homeostasis. Arthritis Rheum..

[11] Leijten J., Georgi N., Moreira Teixeira L., van Blitterswijk C.A., Post J.N., Karperien M. (2014). Metabolic programming of mesenchymal stromal cells by oxygen tension directs chondrogenic cell fate. Proc. Natl. Acad. Sci. USA.

[12] Enomoto H., Enomoto-Iwamoto M., Iwamoto M., Nomura S., Himeno M., Kitamura Y., Kishimoto T., Komori T. (2000). Cbfa1 is a positive regulatory factor in chondrocyte maturation. J. Biol. Chem..

[13] Orfanidou T., Iliopoulos D., Malizos K.N., Tsezou A. (2009). Involvement of SOX9 and FGF-23 in RUNX2 regulation in osteoarthritic chondrocytes. J. Cell. Mol. Med..

[14] Zheng Q., Zhou G., Morello R., Chen Y., Garcia-Rojas X., Lee B. (2003). Type X collagen gene regulation by RUNX2 contributes directly to its hypertrophic chondrocyte-specific expression in vivo. J. Cell Biol..

[15] Von der Mark K., Frischholz S., Aigner T., Beier F., Belke J., Erdmann S., Burkhardt H. (1995). Upregulation of type X collagen expression in osteoarthritic cartilage. Acta Orthop. Scand. Suppl..

[16] Zhou J., Wei X., Wei L. (2014). Indian hedgehog, a critical modulator in osteoarthritis, could be a potential therapeutic target for attenuating cartilage degeneration disease. Connect. Tissue Res..

[17] Wei F., Zhou J., Wei X., Zhang J., Fleming B.C., Terek R., Pei M., Chen Q., Liu T., Wei L. (2012). Activation of Indian hedgehog promotes chondrocyte hypertrophy and upregulation of MMP-13 in human osteoarthritic cartilage. Osteoarthr. Cartil..

[18] Dell’Accio F., De Bari C., El Tawil N.M., Barone F., Mitsiadis T.A., O'Dowd J., Pitzalis C. (2006). Activation of WNT and BMP signaling in adult human articular cartilage following mechanical injury. Arthritis Res. Ther..

[19] Pritzker K.P., Gay S., Jimenez S.A., Ostergaard K., Pelletier J.P., Revell P.A., Salter D., van den Berg W.B. (2006). Osteoarthritis cartilage histopathology: Grading and staging. Osteoarthr. Cartil..

[20] Bi W., Deng J.M., Zhang Z., Behringer R.R., de Crombrugghe B. (1999). SOX9 is required for cartilage formation. Nat. Genet..

[21] Zhao Q., Eberspaecher H., Lefebvre V., de Crombrugghe B. (1997). Parallel expression of SOX9 and Col2a1 in cells undergoing chondrogenesis. Dev. Dyn..

[22] Leijten J.C., Bos S.D., Landman E.B., Georgi N., Jahr H., Meulenbelt I., Post J.N., van Blitterswijk C.A., Karperien M. (2013). GREM1, FRZB and DKK1 mRNA levels correlate with osteoarthritis and are regulated by osteoarthritis-associated factors. Arthritis Res. Ther..

[23] Mariani E., Pulsatelli L., Facchini A. (2014). Signaling pathways in cartilage repair. Int. J. Mol. Sci..

[24] Almonte-Becerril M., Navarro-Garcia F., Gonzalez-Robles A., Vega-Lopez M.A., Lavalle C., Kouri J.B. (2010). Cell death of chondrocytes is a combination between apoptosis and autophagy during the pathogenesis of Osteoarthritis within an experimental model. Apoptosis.

[25] Pascual Garrido C., Hakimiyan A.A., Rappoport L., Oegema T.R., Wimmer M.A., Chubinskaya S. (2009). Anti-apoptotic treatments prevent cartilage degradation after acute trauma to human ankle cartilage. Osteoarthr. Cartil..

[26] Buckwalter J.A., Saltzman C., Brown T. (2004). The impact of osteoarthritis: Implications for research. Clin. Orthop. Relat. Res..

[27] Brew C.J., Clegg P.D., Boot-Handford R.P., Andrew J.G., Hardingham T. (2010). Gene expression in human chondrocytes in late osteoarthritis is changed in both fibrillated and intact cartilage without evidence of generalised chondrocyte hypertrophy. Ann. Rheum. Dis..

[28] Haag J., Gebhard P.M., Aigner T. (2008). SOX gene expression in human osteoarthritic cartilage. Pathobiology.

[29] Aigner T., Fundel K., Saas J., Gebhard P.M., Haag J., Weiss T., Zien A., Obermayr F., Zimmer R., Bartnik E. (2006). Large-scale gene expression profiling reveals major pathogenetic pathways of cartilage degeneration in osteoarthritis. Arthritis Rheum..

[30] Aigner T., Gebhard P.M., Schmid E., Bau B., Harley V., Poschl E. (2003). SOX9 expression does not correlate with type II collagen expression in adult articular chondrocytes. Matrix Biol..

[31] Ma B., Leijten J.C., Wu L., Kip M., van Blitterswijk C.A., Post J.N., Karperien M. (2013). Gene expression profiling of dedifferentiated human articular chondrocytes in monolayer culture. Osteoarthr. Cartil..

[32] Marlovits S., Hombauer M., Truppe M., Vecsei V., Schlegel W. (2004). Changes in the ratio of type-I and type-II collagen expression during monolayer culture of human chondrocytes. J. Bone. Jt. Surg. Br..

[33] Casagrande D., Stains J.P., Murthi A.M. (2015). Identification of shoulder osteoarthritis biomarkers: Comparison between shoulders with and without osteoarthritis. J. Shoulder Elbow Surg..

[34] Aigner T., Zien A., Gehrsitz A., Gebhard P.M., McKenna L. (2001). Anabolic and catabolic gene expression pattern analysis in normal versus osteoarthritic cartilage using complementary DNA-array technology. Arthritis Rheum..

[35] Gebhard P.M., Gehrsitz A., Bau B., Soder S., Eger W., Aigner T. (2003). Quantification of expression levels of cellular differentiation markers does not support a general shift in the cellular phenotype of osteoarthritic chondrocytes. J. Orthop. Res..

[36] Lane N.E., Nevitt M.C., Lui L.Y., de Leon P., Corr M. (2007). Wnt signaling antagonists are potential prognostic biomarkers for the progression of radiographic hip osteoarthritis in elderly Caucasian women. Arthritis Rheum..

[37] Voorzanger-Rousselot N., Ben-Tabassi N.C., Garnero P. (2009). Opposite relationships between circulating Dkk-1 and cartilage breakdown in patients with rheumatoid arthritis and knee osteoarthritis. Ann. Rheum. Dis..

[38] Honsawek S., Tanavalee A., Yuktanandana P., Ngarmukos S., Saetan N., Tantavisut S. (2010). Dickkopf-1 (Dkk-1) in plasma and synovial fluid is inversely correlated with radiographic severity of knee osteoarthritis patients. BMC Musculoskelet. Disord..

[39] Lories R.J., Peeters J., Bakker A., Tylzanowski P., Derese I., Schrooten J., Thomas J.T., Luyten F.P. (2007). Articular cartilage and biomechanical properties of the long bones in Frzb-knockout mice. Arthritis Rheum..

[40] Huebner J.L., Johnson K.A., Kraus V.B., Terkeltaub R.A. (2009). Transglutaminase 2 is a marker of chondrocyte hypertrophy and osteoarthritis severity in the Hartley guinea pig model of knee OA. Osteoarthr. Cartil..

[41] Kim D.Y., Taylor H.W., Moore R.M., Paulsen D.B., Cho D.Y. (2003). Articular chondrocyte apoptosis in equine osteoarthritis. Vet. J..

[42] Kamekura S., Kawasaki Y., Hoshi K., Shimoaka T., Chikuda H., Maruyama Z., Komori T., Sato S., Takeda S., Karsenty G. (2006). Contribution of runt-related transcription factor 2 to the pathogenesis of osteoarthritis in mice after induction of knee joint instability. Arthritis Rheum..

[43] Van der Kraan P.M., van den Berg W.B. (2012). Chondrocyte hypertrophy and osteoarthritis: Role in initiation and progression of cartilage degeneration?. Osteoarthr. Cartil..

[44] Gouttenoire J., Valcourt U., Ronziere M.C., Aubert-Foucher E., Mallein-Gerin F., Herbage D. (2004). Modulation of collagen synthesis in normal and osteoarthritic cartilage. Biorheology.

[45] Boos N., Nerlich A.G., Wiest I., von der Mark K., Ganz R., Aebi M. (1999). Immunohistochemical analysis of type-X-collagen expression in osteoarthritis of the hip joint. J. Orthop. Res..

[46] Blom A.B., Brockbank S.M., van Lent P.L., van Beuningen H.M., Geurts J., Takahashi N., van der Kraan P.M., van de Loo F.A., Schreurs B.W., Clements K. (2009). Involvement of the Wnt signaling pathway in experimental and human osteoarthritis: Prominent role of Wnt-induced signaling protein 1. Arthritis Rheum..

[47] Corr M. (2008). Wnt-β-catenin signaling in the pathogenesis of osteoarthritis. Nat. Clin. Pract. Rheumatol..

[48] Shen J., Li S., Chen D. (2014). TGF-β signaling and the development of osteoarthritis. Bone Res..

[49] Papathanasiou I., Malizos K.N., Tsezou A. (2012). Bone morphogenetic protein-2-induced Wnt/β-catenin signaling pathway activation through enhanced low-density-lipoprotein receptor-related protein 5 catabolic activity contributes to hypertrophy in osteoarthritic chondrocytes. Arthritis Res. Ther..

[50] Blaney Davidson E.N., Vitters E.L., van der Kraan P.M., van den Berg W.B. (2006). Expression of transforming growth factor-β (TGFβ) and the TGFβ signalling molecule SMAD-2P in spontaneous and instability-induced osteoarthritis: Role in cartilage degradation, chondrogenesis and osteophyte formation. Ann. Rheum. Dis..

[51] Nakase T., Miyaji T., Tomita T., Kaneko M., Kuriyama K., Myoui A., Sugamoto K., Ochi T., Yoshikawa H. (2003). Localization of bone morphogenetic protein-2 in human osteoarthritic cartilage and osteophyte. Osteoarthr. Cartil..

[52] Schmal H., Salzmann G.M., Langenmair E.R., Henkelmann R., Sudkamp N.P., Niemeyer P. (2014). Biochemical characterization of early osteoarthritis in the ankle. Sci. World J..

[53] Archer C.W., Francis-West P. (2003). The chondrocyte. Int. J. Biochem. Cell Biol..

[54] Zamli Z., Sharif M. (2011). Chondrocyte apoptosis: A cause or consequence of osteoarthritis?. Int. J. Rheum. Dis..

[55] Landman E.B., Miclea R.L., van Blitterswijk C.A., Karperien M. (2013). Small molecule inhibitors of WNT/β-catenin signaling block IL-1β- and TNFα-induced cartilage degradation. Arthritis Res. Ther..

