# Supplementary material for: Correlation between Gene Expression and Osteoarthritis Progression in Human
Source: Int J Mol Sci. 2016 Jul 14;17(7):1126. doi: 10.3390/ijms17071126 (PMC4964500; doi:10.3390/ijms17071126)
Supplement: Supplementary file 1 [file ijms-17-01126-s001.pdf]

# Supplementary Materials: Correlation between Gene Expression and Osteoarthritis Progression in Human

**Leilei Zhong, Xiaobin Huang, Marcel Karperien and Janine N. Post**

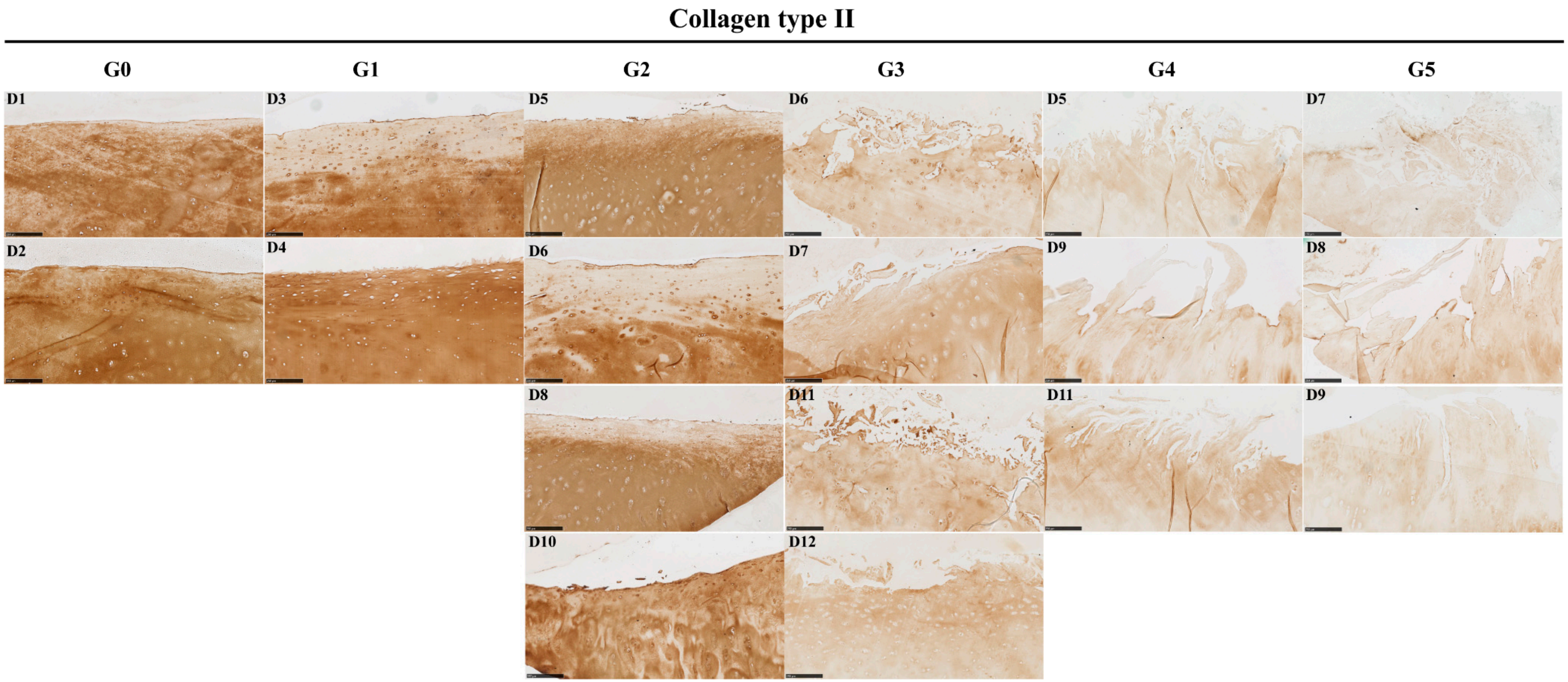


**Figure S1.** The protein expression of collagen type II was detected by IHC in each donor (scale bar: 250 µm). Images were taken using the Nanozoomer. G0, G1, G2, G3, G4, G5 = Grade 0, Grade 1, Grade 2, Grade 3, Grade 4, Grade 5; D1–D12 = Donors 1–12.

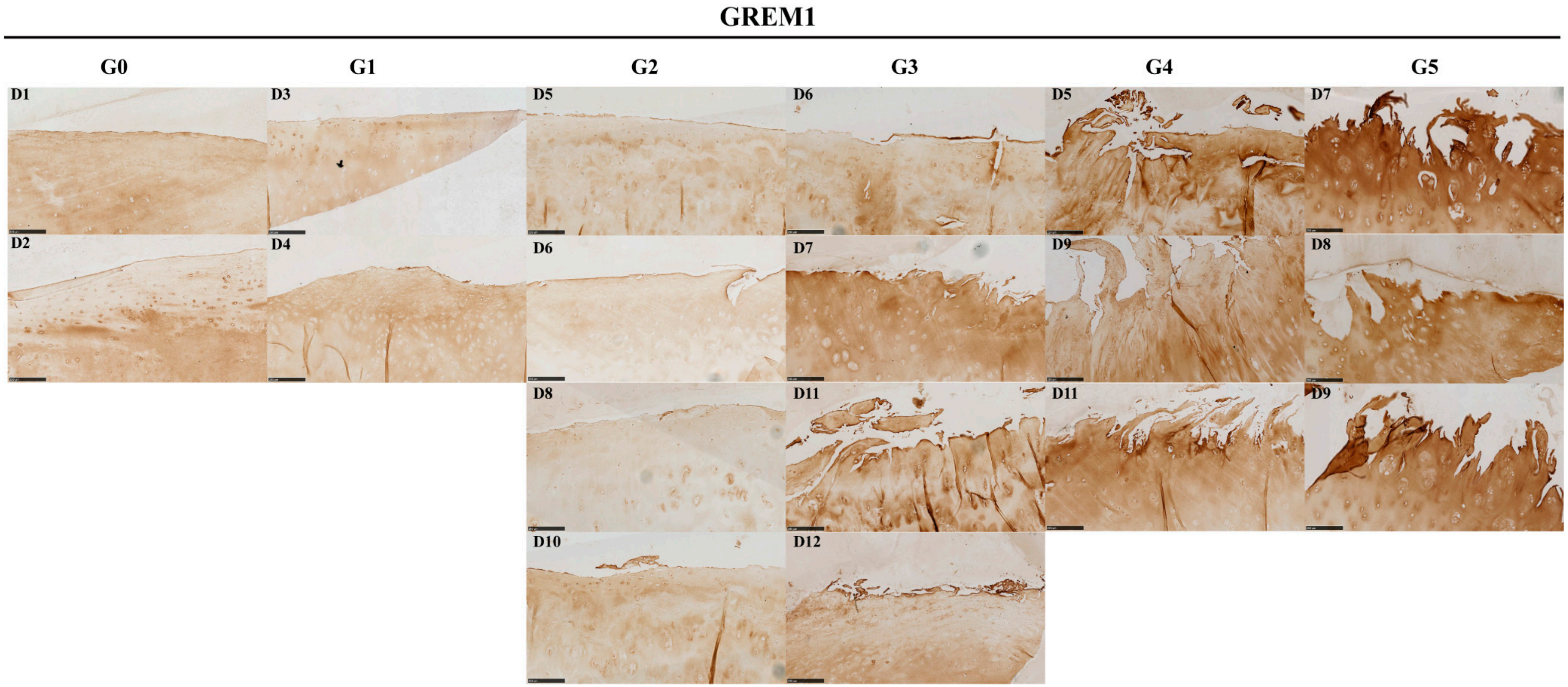


**Figure S2.** The protein expression of GREM1 was detected by IHC in each donor (scale bar: 250 µm). Images were taken using the Nanozoomer. G0, G1, G2, G3, G4, G5 = Grade 0, Grade 1, Grade 2, Grade 3, Grade 4, Grade 5; D1–D12 = Donors 1–12.

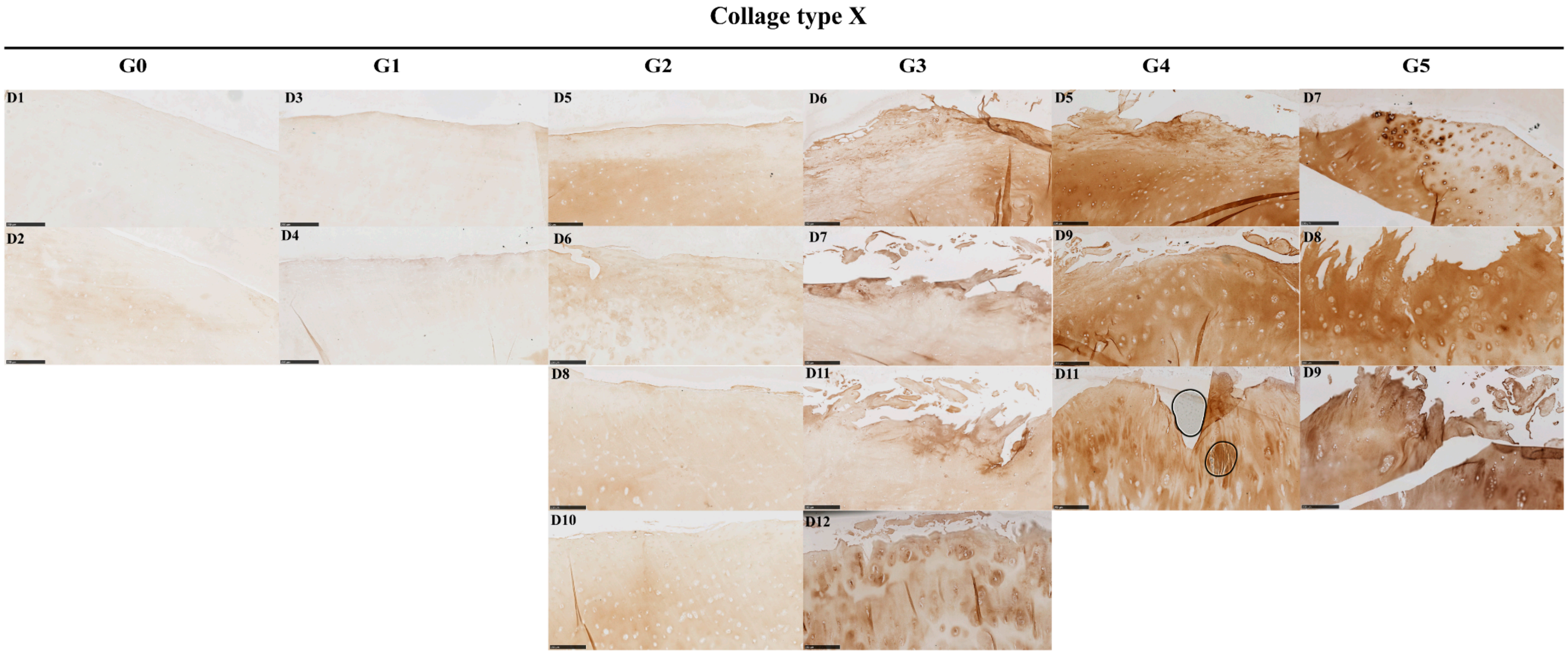


**Figure S3.** The protein expression of collagen type X was detected by IHC in each donor (scale bar: 250 μm). Images were taken using the Nanozoomer. G0, G1, G2, G3, G4, G5 = Grade 0, Grade 1, Grade 2, Grade 3, Grade 4, Grade 5; D1–D12 = Donors 1–12.

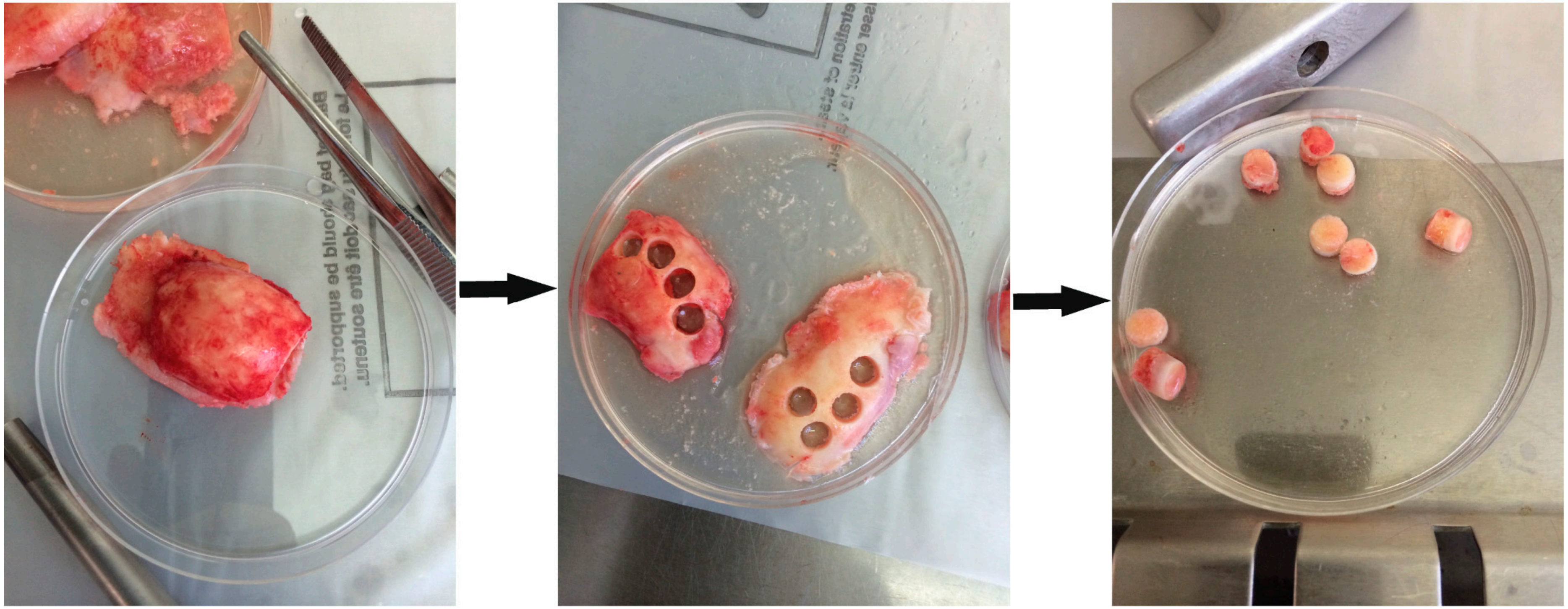

**Figure S4.** Articular cartilage was obtained from patients. The cartilage samples with subchondral bone were punched aseptically using a steel punch. Doughnut-shaped cartilage cylinders were obtained. Subsequently, the subchondral bone was removed.

**Table S1.** Pearson correlations of all tested genes. Correlation coefficient determined by Pearson's correlation analysis * $p < 0.05$, ** $p < 0.01$, *** $p < 0.001$.

| $R^2$ | *SOX9* | *ACAN* | *COL2A1* | *DKK1* | *FRZB* | *GREM1* | *COL10A1* | *IHH* | *RUNX2* | *AXIN2* |
|---|---|---|---|---|---|---|---|---|---|---|
| *SOX9* | – | – | – | – | – | – | – | – | – | – |
| *ACAN* | 0.87 * | – | – | – | – | – | – | – | – | – |
| *COL2A1* | 0.89 * | 0.96 | – | – | – | – | – | – | – | – |
| *DKK1* | 0.61 | 0.76 | 0.82 | – | – | – | – | – | – | – |
| *FRZB* | 0.72 | 0.94 ** | 0.86 * | 0.82 * | – | – | – | – | – | – |
| *GREM1* | −0.75 | −0.57 | −0.57 | −0.34 | −0.41 | – | – | – | – | – |
| *COL10A1* | −0.72 | −0.55 | −0.55 | −0.36 | −0.42 | 0.99 *** | – | – | – | – |
| *IHH* | −0.97 ** | −0.91 * | −0.91 * | −0.63 | −0.78 | 0.83 * | 0.81 | – | – | – |
| *RUNX2* | −0.78 | −0.65 | −0.65 | −0.77 | −0.60 | 0.99 ** | 0.99 * | 0.91 | – | – |
| *AXIN2* | −0.91 * | −0.68 | −0.68 | −0.46 | −0.54 | 0.93 ** | 0.93 * | 0.91 * | 0.95 * | – |
| *BMP2* | 0.34 | −0.16 | −0.16 | −0.28 | −0.38 | −0.39 | −0.37 | −0.19 | −0.70 | −0.48 |

**Table S2.** Patient's information. Cartilage was obtained from 12 patients (68 ± 6 years) with OA undergoing total knee replacement surgery. Patient's information, including age and gender (male (M) and female (F)), is indicated in this table. In addition, some cartilage specimens with different grades were collected from the same patient and cartilage specimens with the same grade were pooled together for RNA isolation. Information is provided for which samples were pooled for RNA extraction.

| **Grade** | | | **0** | **1** | **2** | **3** | **4** | **5** |
|---|---|---|---|---|---|---|---|---|
| **Donor Information** | | | | | | | | |
| **Donor Number** | **Age** | **Gender** | **Pooled RNA Sample (*n* = 2)** | **Pooled RNA Sample (*n* = 2)** | **Pooled RNA Sample (*n* = 4)** | **Pooled RNA Sample (*n* = 4)** | **Pooled RNA Sample (*n* = 3)** | **Pooled RNA Sample (*n* = 3)** |
| 1 | 60 | F | × | – | – | – | – | – |
| 2 | 62 | M | × | – | – | – | – | – |
| 3 | 79 | M | – | × | – | – | – | – |
| 4 | 65 | F | – | × | – | – | – | – |
| 5 | 67 | M | – | – | × | – | × | – |
| 6 | 66 | F | – | – | × | × | – | – |
| 7 | 78 | M | – | – | – | × | – | × |
| 8 | 64 | F | – | – | × | – | – | × |
| 9 | 69 | F | – | – | – | – | × | × |
| 10 | 65 | M | – | – | × | – | – | – |
| 11 | 67 | F | – | – | – | × | × | – |
| 12 | 71 | M | – | – | – | × | – | – |

**Table S3.** Forward and reverse primers used for quantitative RT-PCR.

| Gene Name | Primer Sequence | Product Size | Annealing Temperature |
|---|---|---|---|
| *GAPDH* | Forward primer: 5′ CGCTCTCTGCTCCTCCTGTT 3′<br>Reverse primer: 5′ CCATGGTGTCTGAGCGATGT 3′ | 81 | 60 |
| *SOX9* | Forward primer: 5′ TGGGCAAGCTCTGGAGACTTC 3′<br>Reverse primer: 5′ ATCCGGGTGGTCCTTCTTGTG 3′ | 98 | 60 |
| *ACAN* | Forward primer: 5′ TTCCCATCGTGCCTTTCCA 3′<br>Reverse primer: 5′ AACCAACGATTGCACTGCTCTT 3′ | 121 | 60 |
| *COL2A1* | Forward primer: 5′ GGCGGGGAGAAGACGCAGAG 3′<br>Reverse primer: 5′ CGCAGCGAAACGGCAGGA 3′ | 129 | 60 |
| *RUNX2* | Forward primer: 5′ GGAGTGGACGAGGCAAGAGTTT 3′<br>Reverse primer: 5′ AGCTTCTGTCTGTGCCTTCTGG 3′ | 133 | 60 |
| *COL10A1* | Forward primer: 5′ GAACTCCCAGCACGCAGAAT 3′<br>Reverse primer: 5′ CCTGTGGGCATTTGGTATCG 3′ | 121 | 60 |
| *IHH* | Forward primer: 5′ GCCATCTCCTCTGCCATGAA 3′<br>Reverse primer: 5′ TGCCAGCCTCAAGGTCTCTA 3′ | 153 | 60 |
| *DKK1* | Forward primer: 5′ AGTACTGCGCTAGTCCCACC 3′<br>Reverse primer:5′ TCCTCAATTTCTCCTCGGAA 3′ | 172 | 60 |
| *FRZB* | Forward primer: 5′ACGGGACACTGTCAACCTCT 3′<br>Reverse primer: 5′ CGAGTCGATCCTTCCACTTC 3′ | 155 | 60 |
| *GREM1* | Forward primer: 5′ GTCACACTCAACTGCCCTGA 3′<br>Reverse primer: 5′ GGTGAGGTGGGTTTCTGGTA 3′ | 375 | 60 |
| *AIXN2* | Forward primer: 5′ AGTGTGAGGTCCACGGAAAC 3′<br>Reverse primer: 5′ CTGGTGCAAAGACATAGCCA 3′ | 103 | 60 |
| *BMP2* | Forward primer: 5′ GCTAGACCTGTATCGCAGGC 3′<br>Reverse primer: 5′ TTTTCCCACTCGTTTCTGGT 3′ | 74 | 60 |
| *COL1A1* | Forward primer: 5′ GTCACCCACCGACCAAGAAACC 3′<br>Reverse primer: 5′ AAGTCCAGGCTGTCCAGGGATG 3′ | 121 | 60 |
